# A review of catechins and their use in atopic dermatitis

**DOI:** 10.1097/itx.0000000000000077

**Published:** 2024

**Authors:** Emma Beagles, Ethan A. Lerner

**Affiliations:** a Department of Dermatology, Harvard Medical School, Boston, MA; b Massachusetts General Hospital, Boston, MA

**Keywords:** Catechins, Green tea, Natural products

## Abstract

Atopic dermatitis (AD) is a chronic inflammatory skin disorder characterized by persistent itching of the skin with its prevalence increasing in the United States. AD has a complex pathogenesis that remains to be fully resolved, though it is shown to involve immune dysregulation and skin barrier dysfunction, with multiple environmental and genetic factors implicated. The interplay between the immune system and environmental exposures can incite immune responses with the release of cytokines, IgE, eosinophils, and mast cells, which trigger symptoms of AD in susceptible patients. There are many therapies used in AD; however, the first-line treatment for flares continues to be corticosteroids. The broad range of therapies available for AD is associated with adverse effects, poor adherence, and financial burden, accentuating the need to assess alternative therapies. A promising alternative therapy is the catechin family, a group of flavonoids with a unique structure that has anti-inflammatory, antimicrobial, antioxidant, and skin barrier modulating properties. In this review, we describe the structure and related properties of catechins, their function, and how they can be utilized in the treatment of AD. Furthermore, we describe limitations associated with the use of catechins and the necessity of further research in this area. The function of catechins has been widely shown to modulate the inflammatory pathway and skin barrier dysfunction that have been implicated in AD and reduce symptoms. While catechins can mitigate symptoms and reduce associated inflammatory markers, further research is required to develop a therapy that retains the beneficial functions of catechins without increasing cytotoxicity.

## Introduction

Atopic dermatitis (AD) is a chronic inflammatory skin disorder with a complex pathogenesis that remains to be fully resolved, though it has been shown to involve immune dysregulation and barrier dysfunction. It is driven by multiple environmental and genetic factors, most commonly impacting children^[1,2]^. Genetic factors include loss-of-function mutations in filaggrin that increase transepidermal water loss, pH alterations, and dehydration^[1–3]^. Transglutaminases, keratins, and intercellular proteins function in maintaining the epidermal barrier^[1,2]^. Skin exposure to environmental allergens can trigger T-helper type 2 (Th2) immune responses that lead to the release of cytokines, including interleukin (IL)-4, IL-5, and IL-13, production of immunoglobulin (Ig)-E and the resultant recruitment and activation of mast cells to trigger an allergic response^[1,2,4,5]^.

The various pathways associated with atopic dermatitis have been studied to identify appropriate treatments. First-line therapy typically includes topical corticosteroids, with topical nonsteroidal agents, calcineurin inhibitors, crisaborole, and ruxolitinib available as other prescription treatment options^[6–9]^. The many medications used in AD have shown improvement in symptoms, though some of the commonly used therapies are associated with damaging side effects. Corticosteroids and calcineurin inhibitors can cause paradoxical skin disease, skin atrophy, and low birth weight infants when used in pregnant women^[7,9,10]^. They can also have associated burning, stinging, and pruritus^[8]^. Aside from side effects, complex treatment regimens, financial burden, infrequent follow-up, phobias of steroid use, and lack of health literacy are associated with poor adherence to these medications^[8,11]^.

There are multiple alternative therapies that have been studied for their use in AD. One alternative is flavonoids which are a class of natural products found in fruits and vegetables^[12]^. Flavonoids include a broad group of compounds that play a variety of biological roles in plants, animals, and bacteria^[12–14]^. Flavonoid compounds can be extracted from plants for use in a variety of diseases. Catechins are a group of flavonoids that have been of recent interest for their potential use in AD. Catechins have anti-inflammatory, antimicrobial, antioxidant, and skin barrier modulatory effects^[4,12,15–19]^. They are considered for use in a variety of skin conditions, such as acne, rosacea, keloids, and wound healing^[18]^. As the prevalence of AD increases throughout the world and particularly in urban settings^[20]^, it is important to determine a wider variety of therapies that can be tailored to what best suits the individual patients.

Given the potential for catechin use in the treatment of AD, we describe the structure and related properties of catechins, their function, and how they may impact AD. Furthermore, we describe limitations associated with the use of catechins and the necessity of further research in this area.

### Structure and properties of catechins

**Figure 1.**
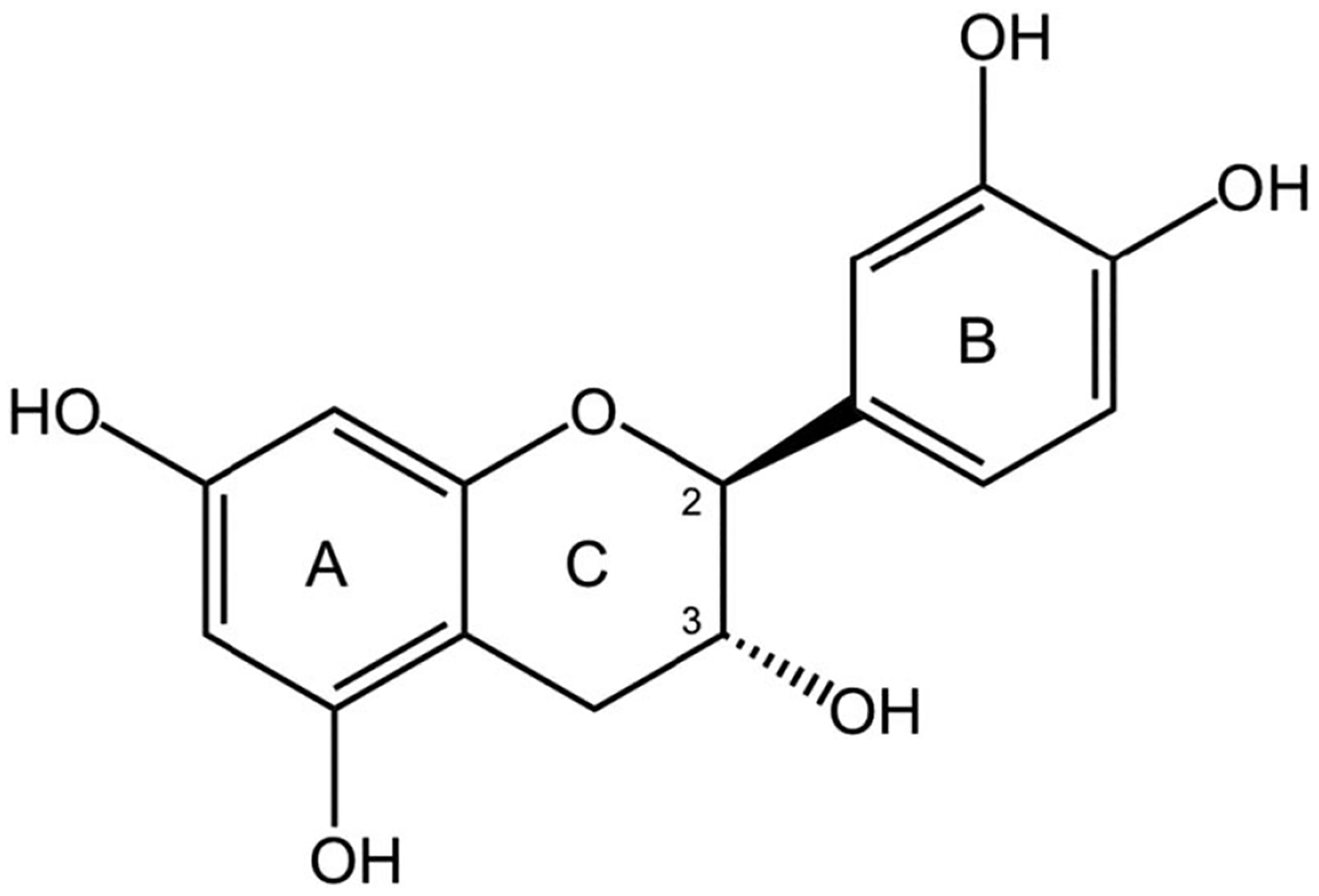
Catechin structure demonstrating benzene rings and a dihydropyran heterocycle with a hydroxyl group on carbon 3.^[21]^

Catechins are within the group of flavanols (flavan-3-ols) commonly found in a variety of fruits, vegetables, and other plants^[12,15]^. The main sources of catechins are green tea, wine, and cocoa. Molecularly, catechins have 2 benzene rings and a dihydropyran heterocycle with a hydroxyl group on carbon 3, as shown in Figure 1^[15,21]^. They are unique among flavonoids as they do not have a double bond between positions 2 and 3 of the carbon ring^[12,15]^. The absence of a double bond between carbons 2 and 3 allows 2 chiral centers that enable the formation of cis and trans stereoisomers. As a result, catechins can make gallic acid conjugates, including epicatechin gallate, epigallocatechin, and epigallocatechin gallate (EGCG) through esterification with gallate groups^[15]^. The conjugates have differing levels of bioactivity, with (−)-EGCG as the most bioactive^[18]^. They are also hydrophilic due to the polarity of the molecule^[22]^. Their overall molecular structure enables the multitude of functions they possess.

### Function of catechins

#### Antimicrobial

The antimicrobial properties of catechins include antiviral, antifungal, antibacterial, and antitoxin functions. Catechins have been shown to reduce infectivity and replication of RNA and DNA viruses^[23]^. In terms of their antifungal activity, catechins enhance the antifungal effects of amphotericin B and fluconazole against *Candida albicans*^[24,25]^. Furthermore, they suppress bacterial growth by disintegrating the microbial membrane, decomposing metabolites, and detaching the cytoplasm. EGCG, in particular, has anti-amyloidogenic features preventing the generation of biofilms in chronically infected wounds^[19]^. In addition, given the negative charge of catechins, they can bind positively charged lipids of bacteria, especially gram-positive bacteria, damaging the lipid bilayer and causing cell death. Catechins also have synergistic effects with antibiotics when used for treating methicillin-resistant *Staphylococcus aureus*. They directly inhibit both the hemolytic activities of *staphylococcus* alpha-toxin and the thermostable direct hemolysin of *Vibrio parahaemolyticus*. In addition, they inhibit Staphylococcal enterotoxin B (SEB), which is an antigen produced by *S. aureus* that activates T-cells and releases inflammatory cytokines^[16]^. Catechins have many functions and mechanisms through which they combat microbes.

#### Antioxidative

Catechins have antioxidant functions and are reported to be among flavones as the most powerful flavonoids for protecting against reactive oxygen species (ROS)^[12]^. Catechins work to directly block ROS, chelate trace elements involved in the production of free radicals, and suppress enzymes needed for ROS generation^[18,19]^. EGCG stabilizes ROS and free radicals by donating a hydrogen atom or electron, making them unable to damage cellular biomolecules. They also suppress the overall production of ROS through interactions with both antioxidant and prooxidant proteins as well as chelation of prooxidant metal ions^[4]^. EGCG has also been shown to have increased expression of heme oxygenase-1 (HO-1), which is an enzyme with cytoprotective properties and ultimately inhibits intracellular ROS production^[26]^.

#### Anti-inflammatory

Catechins target a variety of pathways and molecules in inflammatory pathways, yielding their strong anti-inflammatory effects. Inflammatory mediators, including nitric oxide, prostaglandins, leukotrienes, and cytokines, are inhibited by catechins^[19]^. Another mechanism through which catechins are anti-inflammatory is through cytokine thymic stromal lymphopoietin (TSLP), which is associated with many allergic diseases. A study published in 2012 examined the role of EGCG on TSLP production, finding that it inhibited the production and mRNA expression of TSLP in human mast cell line (HMC-1) cells^[5]^. They determined that EGCG inhibited nuclear factor-κB (NFKB) luciferase activity and caspase-1 activation, indicating that ECGC blocks the caspase-1/NFkB signal cascade in mast cells^[5]^. EGCG has also been shown to suppress interferon-γ (IFN-γ)-mediated priming in human epidermal keratinocytes thus attenuating interleukin-1β (IL-1β) secretion^[27]^. A 2008 study assessing AD-like lesions in NC/Nga mice reported that ECGC diminished the expression of IgE, macrophage migration inhibitory factor (MIF), and other cytokines, including tumor necrosis factor-α (TNF-α), IFN-γ, IL-2, and IL-12^[17]^. Catechins’ impact on factors that are implicated in inflammatory processes enables their use in inflammatory diseases, like AD.

#### Epidermal functions

Catechins play a crucial role in strengthening the skin barrier, particularly at the epidermal level. Proper functioning of the skin barrier relies on various proteins such as filaggrin, loricrin, involucrin, keratins, claudin, and occludin^[1,28]^. Catechins exert their effects on these proteins through different mechanisms, ultimately enhancing skin barrier integrity. It has been demonstrated that catechins can increase the levels of filaggrin, loricrin, keratins, involucrin, and occludin^[19,28,29]^. The production of these proteins can be influenced further by Th2 cytokines, wherein their overexpression reduces protein production^[1]^. By mitigating the Th2 immune response, catechins further contribute to improving the skin barrier. Moreover, catechins have been found to reduce transepidermal water loss, a key indicator of skin barrier function, thereby emphasizing their significance in skin barrier integrity^[30]^.

### Catechins in atopic dermatitis

Identifying therapies that address multiple components of the complex pathogenesis of AD is crucial, making catechins a promising alternative therapy given their antimicrobial, antioxidant, anti-inflammatory, and skin barrier modulatory properties. The various functions of catechins combine to exert an immunomodulatory effect that may reduce symptoms of AD.

#### Epidermal pathogens

Cutaneous infection and microbial dysbiosis are contributors to the symptoms of AD. A variety of pathogens, including *S. aureus*, *Malassezia*, and certain viruses can exacerbate symptoms or cause overlying infections. During a flare of AD, patients may have reduced bacterial diversity of the skin flora, with increased rates of *Staphylococcal* species. Patients with more severe disease tend to have *S. aureus* predominance, which can induce T-cell expansion, upregulate proinflammatory cytokines including TSLP, IL-4, IL-12, and IL22, and stimulate mast cell degranulation^[1]^.

While both *S. aureus* and *Malassezia* are frequently detected on the affected skin of patients with AD as part of the normal skin flora, they are also associated with superinfections and worse symptoms of AD^[16,30]^. A 2012 study treated patients with *Malassezia*-associated AD with green tea bath therapy. Within the study, they assessed the scoring atopic dermatitis (SCORAD) index, visual analog scale for pruritus, transepidermal water loss, and eosinophil counts before and after treatment with a green tea extract bath. They found that all patients had improvement in mean SCORAD, visual analog scale, and decreased mean serum eosinophil counts after treatment. The clinical improvements noted in their study occurred in the popliteal and antecubital fossae^[30]^. As *Malassezia* is often present on the face and upper body, it would be of interest to know if there was a specific impact in these areas, too. The therapy itself was tolerated well by the patients and consisted of a 30-minute bath in 700 mL green tea extract in 150 L filtered tap water 3 times weekly for 4 weeks^[30]^. As *S. aureus* is also implicated in AD, assessing the utility of catechins against the aggravating factors of *staphylococcal* superantigens (SsAgs) is essential. An SsAg of interest is staphylococcal enterotoxin B (SEB), which induces the production of inflammatory cytokines, including TNF-alpha, IFN-y, and IL-4^[16,30]^. When assessing the production of cytokines and SsAg-induced T-cell activation in mice, catechins were able to neutralize SEB and inhibit the SEB-induced immunologic reaction^[16]^. The combined functions of catechins were able to reduce the clinical severity of AD as well as the underlying immune response related to skin pathogens.

#### Immunomodulation

Ultimately, the properties of catechins work in an immunomodulatory fashion, making them a potential therapy for AD. With the underlying pathogenesis of AD relating largely to immune dysregulation and inflammation, focusing on these pathways leads to symptom improvement.

First, a key factor in AD pathogenesis is oxidative stress, which induces proinflammatory cytokine production and triggers a cutaneous inflammatory process that ends in chronic skin inflammation^[4]^. As catechins can inhibit ROS production and stabilize them^[4,18,19,26]^, they may be used to reduce intracellular oxidative stress and thus reduce allergic inflammation^[31]^. NC/Mga mice are used widely as animal models with relevant characteristics for AD, including elevated IgE, chronic skin dryness, and severe pruritus, thus allowing better assessment of therapeutic out comes. A study assessing the effects of topical administration of catechins in NC/Nga mice demonstrated that the antioxidative properties of catechins reduced mRNA and protein expression of cyclooxygenase-2 (COX-2) and inducible nitric oxide synthase (iNOS) in the skin tissues. In addition, it resulted in reduced serum eosinophils, IgE, and Th2 cytokines and ultimately showed improvement in AD lesions^[31]^. Another study formulating epigallocatechin-3-gallate nanoparticles demonstrated improved AD skin lesions by reducing both systemic and local oxidative stress through increased activity of antioxidative enzymes. Downstream of oxidative stress falls the mitogen-activated protein kinases pathway (MAPK), which leads to activation of TNF-alpha and regulates cell death. Through blocking the activation of the MAPK pathway, the regulation of cell death was restored^[32]^. Catechins have also been shown to have reduction of myeloperoxidase activity, inhibiting neutrophilic infiltration through ROS suppression, which has been shown to be important in induction of contact dermatitis^[19]^. By blocking different path ways that are associated with oxidative stress, catechins can prevent the inflammatory response from occurring.

In addition to oxidative stress, catechins also impact cytokines and the resulting inflammatory process. Multiple studies over recent years have utilized different methods of administering catechins to determine the potential impact on disease severity for AD. Given the multitude of cytokines, chemokines, and inflammatory cells that are involved in the pathogenesis of AD, researchers have used these factors to assess treatment response. Again utilizing NC/Nga mice, AD-like skin lesions were induced to monitor clinical severity score, ear thickness, histology, and cytokine expression in response to topical application of 2.5% EGCG. This revealed suppression of MIF, T-helper 1 cytokines, and IgE, coinciding with improved histologic grading and reduced clinical severity scores based on itch, erythema, edema, excoriation, and dryness^[17]^. A 2018 study similarly found that topical catechins reduced inflammatory cytokines and showed improved clinical symptoms of dermatitis. Notably, they identified that catechins inhibit early leukocytic infiltration, which glucocorticoid treatment did not affect^[19]^. Catechins also disrupt the priming of both local and systemic Th2 responses by preventing the formation of TSLP, which is prevalent within keratinocytes of AD skin lesions. EGCG directly inhibits the production and mRNA expression of TSLP in mast cells therefore having the capacity to reduce inflammation and potentially treat atopic diseases like AD^[5]^. Two specific cytokines that catechins have been shown to effect are IL-13 and IL-31. Both cytokines have been implicated in the pathogenesis of AD and are involved in the Th2 immune response. In addition to affecting these 2 cytokines through their impact on the Th2 response, catechins also decreased gene expression of IL-31 and reduced production of IL-13 in sensitized mice^[4,31,33,34]^.

Many other recent studies have focused on the effects of catechins against AD-like skin lesions. One study of note utilized NC/Nga mice treated with a green tea extract of which catechins accounted for 75%–80% of the active compounds. In this work, green tea extracts were treated with tannase, an enzyme that hydrolyzes esters and lateral bonds of tannins, so as to increase the radical-scavenging activity of the catechins. The tannasedigested extracts were administered orally to the NC/Nga mice. Treated animals were found to have a reduction in AD-related symptoms, lower histamine, IgE, and cytokine levels. These mice also had reduced epidermal hyperplasia and fewer inflammatory cells, with significantly fewer mast cells after treatment^[35]^.

#### Skin barrier modulation

Aside from immune dysregulation, barrier dysfunction significantly contributes to the pathogenesis of atopic dermatitis (AD). Genetic factors, such as loss-of-function mutations in filaggrin, are recognized as key players in AD development^[1–3]^. Furthermore, certain cytokines, including IL-13 and IL-31, each implicated in AD pathogenesis, can suppress the production of crucial epidermal barrier proteins, including filaggrin, keratins, loricrin, and involucrin. In addition, the overexpression of Th2 cytokines has been linked to decreased levels of loricrin and involucrin in AD^[1]^. Given this interplay, the immunomodulatory effects of catechins, as discussed above, particularly on Th2 responses, offer promise in enhancing the skin barrier to aid in the therapy of AD.

Previous investigations into the impact of catechins on skin barrier function have demonstrated improvements in transepidermal water loss and reduced dryness in AD-like skin lesions in NC/Nga mice^[17,28]^. Together, these observations underscore the significance of the skin barrier in AD and highlight the therapeutic potential of catechins in managing the condition.

#### Limitations, current studies, and future implications

While research exemplifies the potential therapeutic benefits of catechins in AD, there are multiple limitations that need to be addressed before more widespread use. Their hydroxyl groups and resulting hydrophilicity prevents adequate penetration of the skin barrier^[19]^. In addition, catechins undergo oxidation reactions when exposed to neutral and basic pH levels such as those in the human gastrointestinal (GI) tract. This reduces bioavailability and increases instability of catechins when taken orally^[32,36]^. Furthermore, high doses are usually required to have any benefits, but at large doses catechins are more unstable and have associated cytotoxicity, hepatotoxicity, nephrotoxicity, and GI tract disorders^[32,37]^. Given the barriers to adequately and safely utilizeing catechins, additional research to combat these challenges is being conducted.

Many strategies to deliver catechins more safely and effectively to treat AD are being developed. To avoid poor palatability of tea intake while also increasing the functionality, tannase was used to improve the extraction of bioactive molecules and increases their radical-scavenging function. This enabled oral administration while retaining therapeutic capability^[35]^. Another mechanism focused on chemical modifications of natural catechins that increase lipid solubility to improve membrane permeability and allow topical administration^[19]^. The use of nanoparticles has also emerged as a potential method to increase water solubility and improve overall stability of catechins^[4,32]^. Both studies demonstrated improvement of AD symptoms after therapy. Notably, the use of a microneedle formulation using L-ascorbic acid as a stabilizer improved the stability of EGCG and was as effective against AD-like skin lesions when administered once weekly when compared with daily topical EGCG with the stabilizer^[4]^. Through structural alterations and additional research, catechins can become a new therapy for AD that may improve compliance while reducing adverse effects associated with current therapies.
